# Temporally Adaptive Sampling: A Case Study in Rare Species Survey Design with Marbled Salamanders (*Ambystoma opacum*)

**DOI:** 10.1371/journal.pone.0120714

**Published:** 2015-03-23

**Authors:** Noah D. Charney, Jacob E. Kubel, Charles S. Eiseman

**Affiliations:** 1 Biology Department, Bryn Mawr College, Bryn Mawr, Pennsylvania, United States of America; 2 Natural Heritage & Endangered Species Program, Division of Fisheries and Wildlife, Westborough, Massachusetts, United States of America; 3 Northfield, Massachusetts, United States of America; Tuscia University, ITALY

## Abstract

Improving detection rates for elusive species with clumped distributions is often accomplished through adaptive sampling designs. This approach can be extended to include species with temporally variable detection probabilities. By concentrating survey effort in years when the focal species are most abundant or visible, overall detection rates can be improved. This requires either long-term monitoring at a few locations where the species are known to occur or models capable of predicting population trends using climatic and demographic data. For marbled salamanders (*Ambystoma opacum*) in Massachusetts, we demonstrate that annual variation in detection probability of larvae is regionally correlated. In our data, the difference in survey success between years was far more important than the difference among the three survey methods we employed: diurnal surveys, nocturnal surveys, and dipnet surveys. Based on these data, we simulate future surveys to locate unknown populations under a temporally adaptive sampling framework. In the simulations, when pond dynamics are correlated over the focal region, the temporally adaptive design improved mean survey success by as much as 26% over a non-adaptive sampling design. Employing a temporally adaptive strategy costs very little, is simple, and has the potential to substantially improve the efficient use of scarce conservation funds.

## Introduction

Improving sampling designs for difficult-to-detect rare species has been the focus of much recent work [1–6]. Imperfect detection is a critical problem when field data are used for a variety of tasks, including analyzing habitat associations, untangling metapopulation dynamics, and managing resources [4, 7–10]. To improve both modeling accuracy and the efficient use of limited conservation resources, researchers seek field methods that maximize detection probability.

Adaptive sampling has long been advocated as an appropriate strategy for surveying species with clumped distributions [11–15]. In adaptive sampling, the number and distribution of sampling units depends on the results of the sampling effort, often in an iterative process. For example, a random set of primary plots in a field may be sampled for a plant species, and then secondary plots will be sampled adjacent to only those primary plots in which the plant is detected. While most adaptive sampling designs have focused on spatially non-random distributions [14], the same approach can be taken towards temporally non-random distributions.

Temporal dependence is a classic issue with detectability [16–18]. Changes in detection probability over time may be caused by regular seasonal changes, or by stochastic demographic and environmental events that vary from year to year. Stochastic events may be uncorrelated across sampling sites, as in a classic metapopulation model, or these events may be correlated, as in years experiencing a severe regional drought [19].

To deal with temporal variability, it is usually advised that sampling be repeated in multiple seasons and in multiple years [17]. However, conducting repeated surveys reduces the overall number of sites that can be visited. Several studies have examined these tradeoffs, and a software package has even been developed to aid researchers in designing efficient sampling protocols [6, 20, 21]. However, these studies focus little on distinguishing the correlated from the uncorrelated variance in detectability. When year-to-year changes in detectability are correlated across all sites in a region, there is an opportunity for management agencies and researchers to invest more resources in the most productive sampling years. Recognizing the importance of correlated stochasticity is particularly important for rare species with large population fluctuations.

In rare species conservation, the primary goal of surveys is often simply to locate previously undocumented populations. While non-detection data is useful in long-term monitoring studies to estimate model parameters, such data may be less useful to resource-management agencies. Often, the mandate of rare species biologists is to find as many occurrences as possible of a focal species for the sake of mapping and protecting representative populations of the species. Given a limited budget, will adopting a temporally adaptive sampling scheme be the best way to achieve that goal?

The design of field surveys is almost always sensitive to predictable changes in detectability over time. For example, frog and bird surveys are typically conducted during breeding season, plant surveys are typically conducted during flowering season, mammal track surveys are often conducted when there is snow on the ground. However, these do not constitute temporally *adaptive* sampling unless initial survey results are used to determine when subsequent surveys will be conducted.

Here, we use a comparison of sampling methods for a rare species with high inter-annual reproductive variance as an opportunity to understand the relative importance of survey technique, seasonal timing, and the year in which surveys are conducted. We then simulate sampling for new locations of this species under a temporally adaptive sampling framework to assess the efficacy of this approach.

## Materials and Methods

### Ethics Statement

Salamander larvae were captured with a dipnet and promptly released without killing, collecting, marking, or otherwise injuring the animals. To avoid unnecessary handling, surveys were terminated at each pond after the first larva was observed. All methods involving vertebrates and field work in this study were approved, overseen, permitted, and carried out through the Natural Heritage and Endangered Species Program of the Massachusetts Division of Fisheries and Wildlife, which reviewed all relevant ethical and legal considerations; permit #119.09SCRA

### Study Organism

In Massachusetts, marbled salamanders (*Ambystoma opacum*) are near the northern limit of their range and are state-listed as Threatened pursuant to the Massachusetts Endangered Species Act (M.G.L. c. 131A) and its implementing regulations (321 CMR 10.00). The species is poorly distributed in the region, and local populations are often isolated. Statewide inventory and monitoring is a critical component of the conservation strategy for marbled salamanders, as new populations are still being discovered and records of known populations must be kept “current” to be eligible for certain regulatory protections. However, as is the case for most wildlife managers, resources for inventory and monitoring efforts are scarce and so cost-effective survey methods are of high importance.

Marbled salamanders are a pond-breeding species with high fidelity to their natal ponds [22]. Adults can live up to 10 years and yearly breeding success is very dependent on pond hydroperiod, which in turn is very sensitive to weather patterns. [23, 24]. Surveying for presence of marbled salamanders can be challenging because, like other pond-breeding salamanders, adult and juvenile individuals live underground in the uplands surrounding breeding ponds during most of the year [26, 27]. Reliable surveys (e.g., drift fences with pitfall traps) for adult and juvenile *A*. *opacum* may be performed during breeding migrations or anticipated dispersal events at certain times of the year, but such surveys require substantial resources and, therefore, are of limited utility for large-scale inventory and monitoring efforts. Given those constraints, surveying natal wetlands for presence of marbled salamander larvae is a common approach.

### Study Area

**Fig 1:**
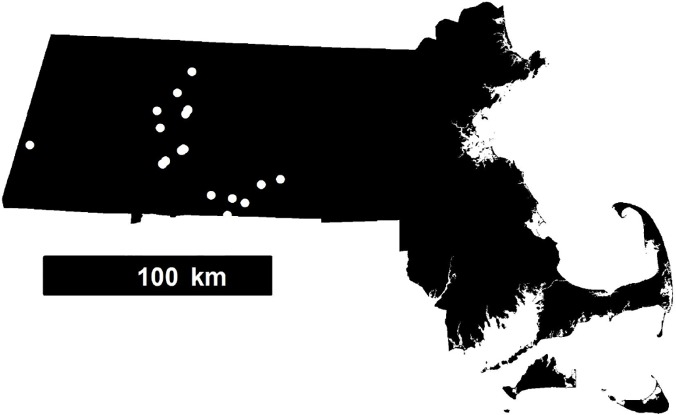
Locations of the 32 study ponds in Massachusetts.

We conducted field surveys at ephemeral wetlands in western Massachusetts (Fig. 1). In addition to our focal species, marbled salamander (*Ambystoma opacum*), we encountered each of the following species in at least 10% of our surveys: spotted salamander (*Ambystoma maculatum*), wood frog (*Lithobates sylvaticus*), green frog (*Lithobates clamitans*), red-spotted newt (*Notophthalmus viridescens*), spring peeper (*Pseudacris crucifer*) and fairy shrimp (*Eubranchipus* sp.). Tree species dominating the wetland canopies included red maple (*Acer rubrum*), red oak (*Qurecus rubra*), eastern hemlock (*Tsuga canadensis*), black gum (*Nyssa sylvatica*), and white pine (*Pinus strobus*). Dominant species of emergent woody vegetation included winterberry (*Ilex verticillata*), highbush blueberry (*Vaccinium corymbosum*), buttonbush (*Cephalanthus occidentalis*), and red-osier dogwood (*Swida sericea*).

### Field Surveys

During two years, 2009 and 2010, we conducted surveys at 32 wetlands known to support breeding by marbled salamanders since 1990, based on records of the Massachusetts Natural Heritage and Endangered Species Program. These ponds represent the known distribution of the species in western and south-central Massachusetts (Fig. 1).

Preferred methods for sampling larval ambystomatid salamanders have been identified through previous research. Fine-mesh (e.g., 3-mm) funnel traps have been found to be more effective than coarse-mesh (e.g., 6-mm) traps in capturing and retaining individuals [28, 29], and capture rates may be improved further by combining aquatic drift fences or similar “leads” with rectangular [30] but not cylindrical [29] traps. Unfortunately, utilization of such methods are not cost-effective for large-scale inventory and monitoring, as materials can be relatively expensive and burdensome to deploy, and the methods require multiple visits to each wetland sampled. Furthermore, nocturnal visual surveys and dipnetting may actually be more effective methods than funnel-trapping [31, 32], although there is not consensus on the subject [33].

In this study, we compared three larval survey methods that require minimal expenditure of financial resources and personnel time: diurnal visual survey, diurnal dipnetting survey, and nocturnal visual survey. Diurnal visual surveys were conducted by visually scanning the wetland from its perimeter for swimming, stratified, or resting larvae during non-rainy, well-lit conditions. Polarized sunglasses were used to reduce glare on the water surface. Diurnal dip-net surveys were conducted by wading into wetlands and sweeping their bottoms and subsurface vegetation with a D-frame net, and examining the contents of each sweep for presence of larvae. Although a variety of depths were sampled, the majority of sweeps were performed at depths of 15–75 cm. Nocturnal visual surveys were conducted by visually scanning the wetland for swimming, stratified, or resting larvae during non-rainy, nighttime hours with the aid of a flashlight. During nocturnal surveys, we observed wetlands both from the perimeter and while wading through them. For all survey types, after marbled salamanders were observed at a pond, species identity was confirmed by catching and photographing characteristic features of one larva. All equipment was sprayed with 10% bleach solution between pond visits to reduce the spread of disease. Field protocols were approved under the Massachusetts Division of Fisheries & Wildlife permit#119.09SCRA.

On each site visit, we surveyed with one of the three methods for the shorter of: 30 min or the amount of time needed to confirm presence of larvae. We sampled wetlands three times each during 2009 and 2010 for a total of six surveys per wetland. In both years, we established three sampling windows of approximately 2 weeks each between 2 April and 26 May. For a given pond, we used a different sampling technique during each window. The order of the three methods used at a given wetland was random, with the condition that the distribution of methods among wetlands was balanced for each sampling window. For a given wetland, the order in which the three methods were used remained the same both years.

We used a survival analysis framework to interpret our field data. The response variable was the time to detection, which corresponded to the survey “survival.” A more efficient sampling technique should yield shorter surveys. In this analysis, we treated surveys with no detections as right-censored data and only included ponds for which marbled salamanders were detected at least once over the course of the entire study. We suspect that ponds where we never found marbled salamanders in any of our surveys likely represent errors in identifying population locations, such as mistaking the breeding pond for one nearby, misidentifications in the historic records, or extinct populations. Such ponds do not fit within our survival framework wherein we assume that all surveys will eventually end in success if carried on indefinitely. While the choice to exclude these ponds has the potential to bias the overall detection efficiencies we report in a positive direction, it should not bias the relative value of each of the methods we are interested in comparing to each other. To evaluate the effect of excluding these ponds in our analyses, we compared estimates from survival analyses using all 32 ponds to the estimates calculated with the smaller set of ponds.

The predictor variables were method (daytime visual, dipnet, nighttime), year (2009, 2010), date window (April 2—April 25, April 24—May 16, May 18—May 26), and observer (N.D.C or C.S.E). We used AIC to select the parameters that formed the best model, including all two-way interaction terms among the possible models considered. For the variables “method,” “year,” and “date window,” we also generated univariate p-values based on a non-parametric resampling analysis. The survival regression provides predicted mean time to detection for each category of each predictor variable. In the non-parametric resampling analysis, our test statistic was the ratio of the maximum and minimum predicted mean time to detection among the categories of a given variable. For each variable, we generated and analyzed 10,000 simulated data sets from our collected data. In the simulated data, all other variables were held fixed as they were originally collected while the values for the focal variable were randomly reassigned to the samples. From the distribution of the test statistic in our simulated data sets, we calculated a p-value as the frequency that we obtained a test statistic more extreme than in our real data. Analyses were conducted using the “survival” package in R 2.10.0 [34, 35]

### Simulation of Temporally Adaptive Sampling

Using the results of our field data we simulated future sampling efforts under a temporally adaptive framework. In each scenario, we assume a budget that allows for a maximum number of pond surveys that must be conducted within a limited number of years. At the beginning of each year, we sample a pre-determined number of “bellwether” ponds, which are long-term monitoring sites with known populations from which we assess the relative larval densities in the focal year. These bellwether pond surveys are deducted from our total budget for surveys. In the same time that it takes to survey an average pond for presence of marbled salamanders, one could easily obtain larval density estimates in a pond with a robust population. We assume that the annual variation of larval densities from the bellwether ponds has been characterized from prior data fit to a beta distribution. From this distribution, we calculate what percentile the current year represents within the beta distribution of the bellwether ponds. We then calculate the probability that all of the remaining years in the study period will have lower reproductive output than the observed output in the current year:

$$p_{best}={F(q,\alpha ,\beta )}^{y-1}$$

Here, *p*_*best*_ is the probability that the current year is the best one we will encounter for the remainder of the study, *F* is the beta cumulative distribution function as described by the beta function coefficients (*α* and *β*) evaluated at the observed reproductive output (q) for this year, and *y* is the number of remaining years in the study, including the current year. If *p*_*best*_ > 0.4, then we allocate all of our remaining budget for surveys to the current year. Otherwise, we conduct no new surveys this year and wait until the following year. In preliminary trials, the simulation outcomes were fairly insensitive to changes in the threshold of *p*_*best*_ at which surveys were conducted. Threshold values ranging from 0.2 to 0.6 yielded very similar results. The outcomes were also very similar when we used a two-threshold criteria. An example of a two-threshold criteria is where all surveys were conducted if *p*_*best*_ was greater than 0.5, but a fraction of the surveys were conducted if *p*_*best*_ was between 0.4 and 0.5. For comparison, we also simulate surveys in a non-adaptive framework where the total number of surveys is allocated equally among the years of the study.

A random subset of the ponds in our simulations represented actual populations, and at those ponds the detectability at each pond varies annually. The variation in detectability is partitioned into a global component and a local component. In each year, we use a single random draw from the beta distribution of detectability to represent the global component of variation for all ponds. The local component is drawn separately for each pond from this same distribution each year. We then perform a weighted average of these terms to obtain the final probability of detection at each pond in each year. The relative weights of the global and local components are described by a correlation term which is fixed at the beginning of each model simulation and can range between 0 and 1. When correlation is 1, all occupied ponds in a given year have the same detectability as described by the global component, whereas when correlation is 0, the global component has no influence on the detection probabilities at each pond.

We fit the beta distribution by combining our field data with a long-term monitoring effort conducted by another research group at a few of the ponds in our sample (K. McGarigal unpublished data). Those researchers have conducted larval abundance surveys annually from 2000 through 2011, except for 2004. We used our 2009 and 2010 multi-pond survey data to convert the long-term abundance estimates to detection probabilities, as follows. At the long-term monitoring sites, larval abundances during the two years of our field surveys (2009 and 2010) were the second lowest and the highest values over the 11-year span of their study. Based on this information, we fit a beta function with a cumulative distribution that matched our observed detection probabilities in 2009 and 2010 at the 9th and 91^st^ percentiles using the “optim” function in the stats package of R.

We began all simulations with a budget sufficient for 400 total pond surveys, including bellwether surveys, to be conducted within 5 years. We let 20% of the ponds encountered represent marbled salamander breeding sites, and we visited each pond only once in order to maximize the number of new sites where they are detected. We varied the number of bellwether ponds from 0 to 10 and the correlation from 0 to 1 at intervals of 0.05. At each parameterization, we conducted 10,000 simulations and then calculated the mean and standard deviation for the number of new marbled salamander detections. All simulations were performed in R 2.10.0.

## Results

**Fig 2:**
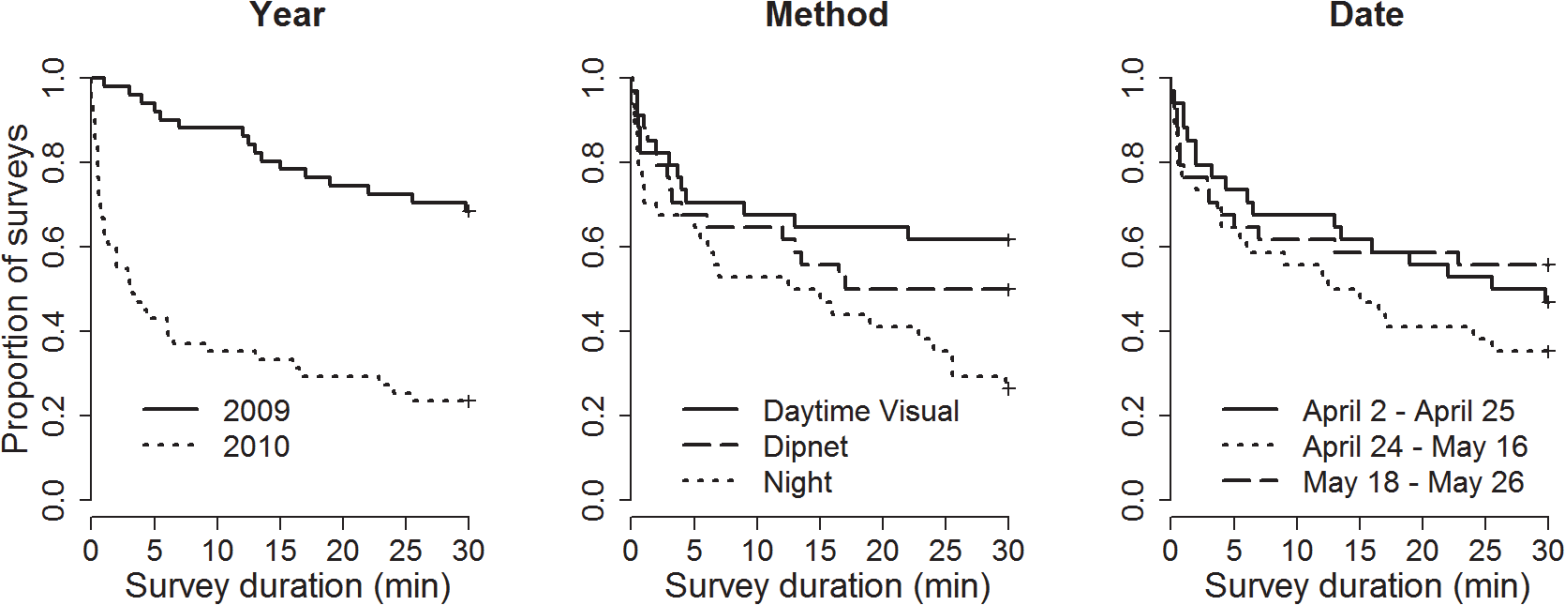
Survival curves depicting the distribution of marbled salamander (*Ambystoma opacum*) time to detection for each category of the three variables: year, method, and date. The categories for which salamanders were detected most readily yielded the shortest surveys, and thus have survival curves that appear lower on the vertical axis.

**Fig 3:**
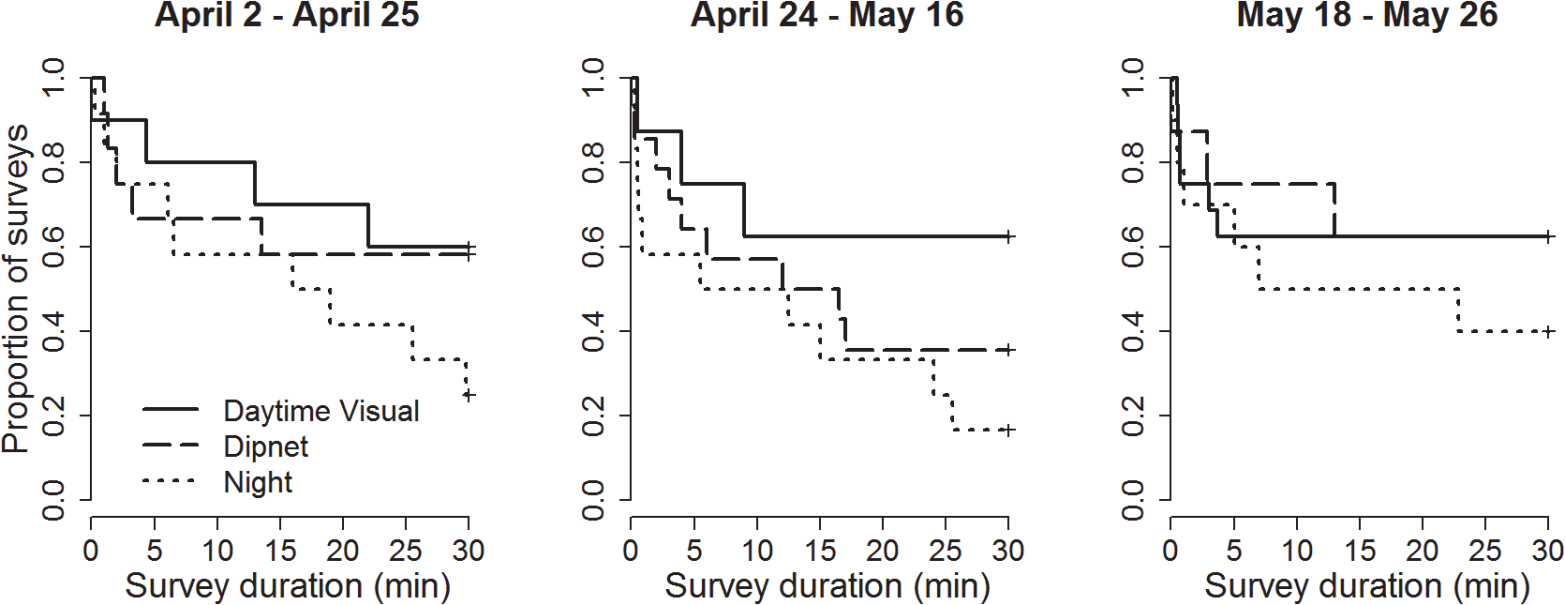
Interaction plot of survival curves depicting the distribution of marbled salamander (*Ambystoma opacum*) time to detection for the each method category during three date windows. The categories for which salamanders were detected most readily yielded the shortest surveys, and thus have survival curves that appear lower on the vertical axis.

Over the course of the field study, we detected marbled salamanders during 55 of our surveys in 17 of the 32 wetlands (S1 Appendix). The model with the lowest AIC contained “year” and “method.” In the survival regression fit, the mean predicted time to detection was significantly lower in 2010 than in 2009 ($\bar{x}$ = 13 min, SE = 3 min for 2010; $\bar{x}$ = 36 min, SE = 3 min for 2009; *p* < 0.0001; Fig. 2). The effect of method was also significant (*p* = 0.01), with the shortest predicted time to detection for nighttime visual surveys ($\bar{x}$ = 18 min, SE = 4 min), intermediate predicted time to detection for dipnet surveys ($\bar{x}$ = 26 min, SE = 4 min), and the longest predicted time to detection for daytime visual surveys ($\bar{x}$ = 31 min, SE = 4 min). The predicted time to detection was shorter during the middle date window ($\bar{x}$ = 20 min, SE = 4 min) than either the early date window ($\bar{x}$ = 27 min, SE = 4 min) or the late date window ($\bar{x}$ = 28 min, SE = 4 min), but this difference was not statistically significant (*p* = 0.2). Visual inspection of the interaction plot between method and date window suggest the potential for dipnet surveys to be most useful during the middle window, although still not as good as night surveys, and no interaction terms were part of the model with the lowest AIC (Fig. 3). In models that included all 32 ponds, as expected, the absolute estimates for time to detection were all approximately twice that of the corresponding estimates in our models where we only included the 17 ponds where marbled salamanders were detected during our study, however, none of the relative patterns changed (S2 Appendix).

**Fig 4:**
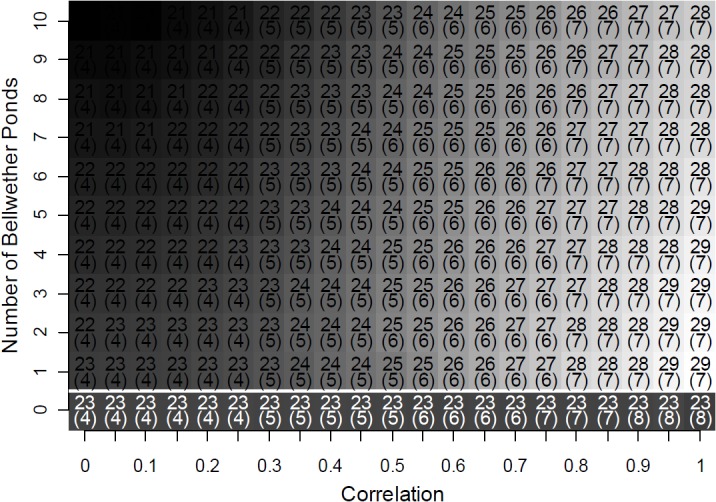
Number of ponds where marbled salamanders (*Ambystoma opacum*) were detected in simulated surveys over 5 years with a budget allocated for 400 total surveys. Bellwether ponds were used to decide whether to survey in a given year. In the simulations below the horizontal white line where no bellwether ponds were used, the surveys were equally divided among each of the years in the study. The correlation term describes the degree to which the annual variance in larval detectability at ponds was synchronized across the study region. For each parameterization of correlation and the number of bellwether ponds, we ran 10,000 simulations. Cell shading and the numbers represent the mean number of ponds where salamanders were detected for each parameterization, with standard deviations below in parentheses.

In our simulated surveys for unknown breeding locations without using bellwether ponds, the mean number of new detections in each model run was 23. Incorporating bellwether ponds increased our mean number of detections to a maximum of 29 when correlation was high, but offered no improvement when correlation was low (Fig. 4). At all levels of correlation, sampling more than one bellwether pond did not improve the number of detections.

## Discussion

Our results demonstrate the potential utility of a temporally adaptive sampling framework. For species with inherently low detectability and substantial population fluctuations correlated across a region, concentrating sampling effort in the most productive years maximizes detection rates. High inter-annual variability in population sizes may be typified by amphibians with dramatic swings in breeding effort [36, 37], but is a well-known phenomenon in the ecology of many organisms including mammals [38], fish [39, 40], invertebrates [41, 42], and plants [43]. While fluctuations in population densities may drive variation in detectability for many organisms, detectability may also vary due to correlated behaviors, such as synchronized flowering and fruiting [44, 45] or altered foraging patterns in response to environmental conditions [46, 47].

When using any adaptive sampling design to make inferences about the whole population, survey units cannot be treated as simple random samples because intentional bias towards the most productive units has been introduced. How one interprets the results of a temporally adaptive sample depends on the goals of the study and the nature of the variability in detectability of the focal organism. If high-detection years are the result of large population sizes, then in those years we may be detecting individuals that have recently colonized areas that do not typically function as suitable habitats and where populations cannot persist, such as sink populations [22]. While information on population abundance may help to determine relative habitat suitability within a set of surveys, studies during high-detection years might overestimate the physical and ecological distribution of the focal species, particularly if the studies are based on presence-absence data. However, such bias would not be expected if high detection years reflect greater visibility due to behaviors like flowering in plants.

The temporally adaptive approach can be modified to suit the goals of inference sought from a particular study. In typical adaptive designs for spatially clustered populations, primary plots are used to estimate the abundance of clusters on the landscape while secondary plots are used to estimate the size of these clusters. A similar strategy could be used in a temporal framework where primary plots are surveyed every year and used to estimate the frequency of population booms while secondary plots are surveyed within productive years to better characterize these population booms.

The sampling strategy in our simulation was sufficient to meet our objective of identifying marbled salamander occurrences for protection under state law. Breeding in a pond represents a visible behavior at the center of a habitat patch that includes the surrounding forest. In years when no larvae are found in a pond, the site may still be occupied. Adults can live for many years in the uplands between breeding events and they usually breed exclusively at the same pond throughout their lifetimes. Thus, for marbled salamanders, annual variance of larval presence likely represents breeding cycles of persistent populations rather than true colonization and extinction events. Furthermore, the operational definition of occupancy from the regulatory standpoint in Massachusetts can include any site where the species has been found within the past 25 years. False positives may be less worrisome than false negatives, as we would rather protect habitat that is occasionally occupied than let occupied habitat be deforested and developed.

The temporally adaptive sampling strategy requires researchers to estimate regional detection probability at the start of each year. One method for achieving this would be to rely on a few long-term monitoring sites with known populations that serve as bellwethers, as we have done in our simulation. This monitoring could be relatively cheap, because our results indicate that very few sites may be needed. In our simulations, a single monitoring location was usually sufficient to reap the full benefits of the adaptive sampling design. Any one pond serves as a good predictor for the other ponds when correlation is high, and when correlation is low there is little benefit in characterizing the global detectability anyway. An alternative approach for estimating population abundances in advance of sampling would be to use a predictive model based on climatic and demographic variables. Using either bellwether ponds or predictive models to guide future surveys has the potential to substantially increase the efficient use of limited agency funds.

The results of our field study suggest that differences in detection probability for marbled salamanders are in fact temporally correlated over our region in western Massachusetts. Despite the observed differences in larval detections among the three sampling methods and the three sampling windows, year was by far the most important predictor for survey success. The overall detection rate jumped from 17% in 2009 to 41% in 2010. The low counts from 2009 can likely be explained by early and heavy rains during fall 2008 that filled breeding wetlands before most females arrived for oviposition, which normally occurs in dry portions of pond basins. We happened to capture a particularly bad year and a particularly good year for marbled salamander reproduction, and so there remains a possibility that, in less extreme years, the choice of survey method could have a greater influence on survey outcome than we observed. However, extreme fluctuations appear to be the norm for this species. The larval abundances in 2010 were the highest on record and 3.5 times greater than the mean observed over the 11 years of monitoring (McGarigal unpublished data). Yet, this year did not appear to be an outlier, as the 2010 abundances were only 1.3 times greater than the second highest year of the study. The coefficient of variance for the annual larval surveys was 1.1, which is consistent with the high catastrophe rates among marbled salamanders observed by Taylor et al. [25].

**Fig 5:**
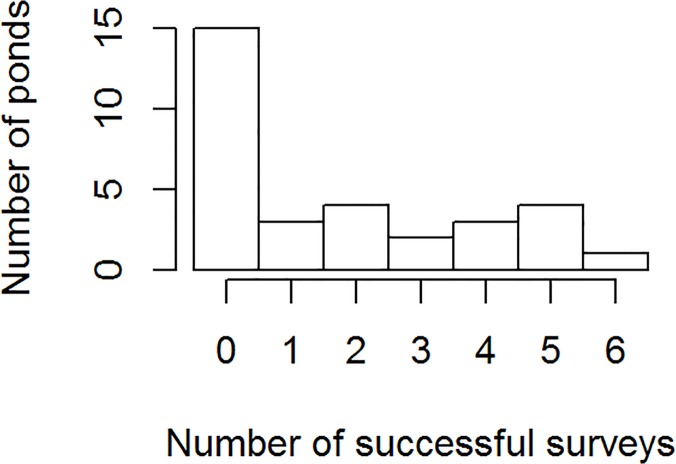
The distribution of successful surveys for marbled salamanders (*Ambysoma opacum*) at 32 wetlands visited 6 times each.

Our study is the first to directly compare the efficacy of diurnal visual, diurnal dipnetting, and nocturnal visual survey methods for determining presence of larval ambystomatid salamanders. Our choice to exclude ponds with no detections may have artificially inflated the overall detection efficiencies that we report. However, the distribution of successful surveys per pond support this approach for the analytical purposes of methods comparison (Fig. 5). Even if marbled salamanders were present at the 15 excluded ponds, these ponds still appear to represent a qualitatively separate set of ponds from the other 17, as only 3 of the 17 ponds had fewer than 2 detections. Variation in wetland area, depth, vegetation, water color and accessibility can all contribute to variation in the ease of detection between ponds. While the best strategy for rapidly finding new populations might be to conduct surveys only at wetlands where detection is easy, it is typically very difficult to determine from GIS data which wetlands will be easier to survey prior to visiting the sites.

While diurnal dipnet and visual surveys were not as successful as nighttime surveys, there are logistical considerations with nighttime surveys that might make them overall less efficient than diurnal surveys. With nighttime surveys, it is more difficult to locate ponds, photograph habitat, ensure landowner permission, maintain the goodwill of neighbors, and recruit field technicians. For these reasons, daytime visits are often required prior to nighttime visits, substantially decreasing the efficiency of the nighttime surveys. Between the two diurnal survey techniques, using a dipnet is much more disruptive to pond life and has the greatest potential for transmitting diseases among ponds. Dipnetting involves continuously churning through the bottom of the wetland flipping up leaves, sediment, vegetation, and anything else that is on the bottom. The net itself typically has many small complicated folds and crevices around the attachment points that are more difficult to clean than boots or waders, thus increasing potential for disease transmission among sites. In ponds with abundant larvae, visual surveys were usually sufficient, and detections would occur within the first few minutes with or without the net. In ponds where the water had low visibility, or the pond had been reduced to mud with no standing water, dipnets allowed detections of larvae that would have likely been missed by visual survey only. The optimum survey method may be a combined approach: survey at night if practical, and if not, spend several minutes surveying visually before sweeping with a dipnet.

In the context of our data set, detailed discussion of the differences among nocturnal visual surveys, diurnal visual surveys, and dipnet surveys for larval salamanders may seem to be splitting hairs. But consider the history of this study as an allegory. Originally, we allocated funding for a single year (2009) to conduct a techniques-comparison study to improve the efficiency of future surveys conducted by the Massachusetts Natural Heritage and Endangered Species Program. An additional goal for this project was to discover previously undocumented populations by surveying ponds not included in the data presented here. While we were aware of the inter-annual variability of marbled salamander reproductive success, a temporally adaptive framework was not part of the initial discussion. Yet, our study has demonstrated that considering the choice of year can be more important than considering the choice of survey technique. Indeed, 2009 would have been an unfortunate year to spend all of our allocated budget for finding new populations. Fortunately, we ultimately adopted a temporally adaptive strategy. The low abundances recorded during the bellwether pond surveys early in the 2009 season prompted us to postpone the 2009 surveys for new populations until 2010. We then had a very productive season in 2010, locating many previously unknown populations in the state.

### Management Implications

A temporally adaptive sampling strategy is intuitive and can be simple to employ. Because few monitoring sites are needed, the initial costs are relatively quite small. Yet the improvements in efficiency are potentially quite large. Our simulations demonstrated improvements in efficiency of up to 26% for marbled salamanders, and even greater gains in efficiency can be expected for species with more extreme population fluctuations. For any species, we recommend that conservation planners assess annual variation in population size and conspicuousness prior to committing resources to survey in a particular year. Efforts should be made both to develop predictive models and to maintain long-term bellwether sites that are representative of the area of interest. Implementing this sampling scheme can be as simple as making a qualitative assessment of population abundance at a known population prior to the start of the field season. Alternatively, managers could maintain quantitative yearly population data and fit these to a beta distribution. Using our equation for *p*_*best*_, managers could then set a threshold which would trigger surveys. Each species and each region may exhibit different degrees of temporal correlation among sites, and care should be taken to understand the spatial dynamics of population fluctuations.

When establishing bellwether sites, several considerations must be taken into account. First, sites should be selected which are conveniently located and at which it is easy to quickly estimate relative visibility of the population in a given year. With the long-term monitoring site for marbled salamanders, obtaining larval density estimates each year is quick. At the largest population, surveys were completed by two people in a single night in under 30 minutes. In this time, they counted nearly 700 larvae in the best year and fewer than 10 larvae in the worst year. Even with just a 5 minute scan from the edge of the pond each year, one could likely categorically discriminate between good years and bad years. Second, there must be sufficient flexibility to allow surveyors to respond to bellwether survey results. This could be achieved with permanent survey staff who have multiple projects that can be re-arranged to accommodate changing field schedules. If survey staff need to be hired as seasonal workers solely for the survey season, bellwether ponds need to be surveyed early enough to allow for hiring within the critical survey window. For some species, it may be possible to predict the quality of an upcoming year based on early indicators from the previous season. With marbled salamanders, it is often possible to conduct larval counts at the long-term sites during fall or winter, well before the start of the spring survey season. Finally, identifying good survey years can be best achieved when sufficient resources and commitment are allocated to regular long term monitoring of bellwether sites, even in years when no other surveys are planned. The cost of long term monitoring could reduce the overall cost effectiveness of the adaptive strategy, particularly if it is not easy to cheaply estimate abundances. If, however, there is already a long term monitoring effort established for the species, as was the case with our study, the prospect for using the existing study as a bellwether may be quite good.

## Supporting Information

S1 Appendix Survey results for marbled salamanders (*Ambysoma opacum*) at 32 wetlands visited 6 times each. (CSV)

S2 Appendix Predicted time to detection of marbled salamanders (*Ambysoma opacum*) for different methods, date windows, or years. Estimates are based on survival analysis for all 32 wetlands in our study (first two columns) or only the 17 wetlands were marbled salamanders were detected at least once (last two columns).
(CSV)
